# Experience of chinese counter-marching nurses with COVID-19 patients’ death in Wuhan: a qualitative study

**DOI:** 10.1186/s12912-023-01270-4

**Published:** 2023-04-27

**Authors:** Zhifang Guo, Kunli Wu, Huibin Shan, Younglee kim, Qilian He

**Affiliations:** 1 College of Nursing, Dali University, Dali, China; 2 Department of Infection Disease, Kunming Third People’s Hospital, Kunming, China; 3 People’s Hospital of Dali Bai Autonomous Prefecture, Dali, China; 4 Department of Nursing, College of Natural Science, California State University, San Bernardino, CA 92407 USA

**Keywords:** COVID-19, Coronavirus, Nurses, Qualitative study, China

## Abstract

**Background:**

The COVID-19 pandemic was occurring worldwide with over a 6.5 million deaths. It’s important to explore the instructions for the global nursing community by identifying the personal coping methods of Chinese nurses in Wuhan to deal with patient deaths.

**Methodology:**

The study used a qualitative conventional content analysis with 14 Chinese Counter-marching nurses. Purposive sampling, snowball sampling, and semi-structured interviews were used for participants and data collection. To assess the quality of the findings, Guba and Lincoln’s criteria for confidence were fulfilled.

**Results:**

The data analysis results in 4 main categories:(1) psychological shocks related to COVID-19 patient’s death; (2) personal psychological adjustment and demands; (3) insights on life and values; (4) Needs for relevant knowledge and skills.

**Conclusions:**

During the outbreak of the epidemic or pandemic, adequate psychological care resources need to be provided to nurses when facing the death of infectious patients, to reduce the negative emotions brought by death. Effective coping strategies should also be formulated to enhance their resilience and promote their professional competence.

## Introduction

The World Health Organization (WHO) declared the COVID-19 viral outbreak a Public Health Emergency of International Concern (PHEIC) on January 31, 2020. COVID-19 is an acute respiratory disease related to the coronavirus. Patients with more severe infections often succumb to renal failure and acute respiratory failure [1, 2]. As of September 18, 2022, WHO reported 609 million confirmed global cases of COVID-19, including 6.5 million deaths in 216 countries [3]. With the increasing number of suspected cases and fatalities, this unpredictable epidemic caused public panic and mental distress at that time [4]. To effectively cope with the COVID-19 outbreak, China’s government implemented rapid and comprehensive public health emergency interventions. Consequently, a large number of healthcare professionals (physicians, nurses, and other healthcare personnel) were urgently recalled for medical rescue support. This entire effort was called “Counter-Marching” and those who supported it were named “the Counter-Marching people”, among whom nurses played a primary role in patient care while directly facing death from COVID-19 patients [5, 6].

During the pandemic, nurses are not only responsible for disseminating policies and providing health education to promote public health but also for delivering high-quality hospice care to severely ill patients. Consequently, healthcare professionals – particularly nurses who have a greater frequency of contact with death [7] – play an essential role in treating patients during outbreaks of infectious diseases and are at the forefront of all efforts to prevent pandemics [8]. Studies on nurses’ experience with dying patients have been conducted in many countries [9–15], revealing that those who provided end-of-life care experienced some positive personal and professional growth [16]. Nevertheless, most nurses find it emotionally challenging to cope with death and dying. The survey showed that 96% of professional healthcare providers would feel at least one type of sadness after a patient’s passing [17]. If these sorrowful emotions remain unresolved, it can lead to job burnout or compassion fatigue among nurses as well as reduce their job satisfaction; this could ultimately affect the quality of end-of-life care given to patients - something which should be closely monitored by nursing researchers and managers.

Though nurses may encounter patient deaths many times throughout their nursing career, during this deadly COVID-19 pandemic, their encounters with dying patients have increased exponentially in a very short period. Overwhelming emotional and psychological distress can emerge in nurses when working with dying patients and repeated exposure to death [18, 19], such as anxiety, distress, and frustration. These negative emotions are one of the stressors that bring mental and psychological problems to clinical nurses. Nurses must be qualified to provide expert care for those at the end of life, including their family members [20], while also maintaining their own well-being [21–23].

Healthcare professionals, especially nurses, are an essential asset for any country. Their mental health and emotional well-being are not only important for the safe and continued treatment of patients but also for containing potential disease outbreaks. Therefore, it is imperative to investigate the entire experience of nurses facing death during the COVID-19 pandemic. Qualitative studies provide a more in-depth understanding of different situations such as the psychological state of nurses during COVID-19 by collecting data based on participants’ real views and experiences. While numerous cross-sectional surveys have provided us with clinical evidence regarding nurses dealing with patient deaths [9, 15, 24], there has been little qualitative research conducted on this topic during the pandemic; thus, further qualitative research is necessary for this field. This qualitative study seeks to explore Chinese nurses’ first-hand accounts of their experiences with COVID-19 patient death. The findings will form a basis from which meaningful and effective interventions can be developed to prepare healthcare workers for caring for infectious disease patients who pass away.

## Methods

### Study design

This study design was based on a conventional content analysis approach. Content analysis is a qualitative research method used to analyze the data, and it is a systematic classification and coding technique aimed at a better understanding of the phenomenon under a study [25, 26]. The investigators used semi-structured interview techniques to gather Chinese nurses’ experiences caring for COVID-19 patients facing death.

### Human subjects review

Before data collection began all study procedures were reviewed and approved by the Medical Ethics Committee of Dali University. Informed consent to participate in this study has been obtained from all participants. The review preparation and process explanation was conducted with similar steps reported in the investigators’ previous study [27].

### Participants and setting

The investigators recruited fourteen Counter-marching nurses from various cities in China to support the crisis in Wuhan. The study had two inclusion criteria: (a) nurses who directly cared for COVID-19 patients; (b) nurses who had personally cared for and experienced the death of at least one COVID-19 patient. The study had one exclusion criterion: a) nurses who declined to participate in the study or were unwilling to be audio-taped during the review. Initially, contact information for four nurses who had worked in COVID-19 wards was obtained by purposive sampling on the recommendation of hospital colleagues; all of them had personally cared for and experienced the death of at least one COVID-19 patient during the pandemic, and were eager to participate in interviews. Then snowball sampling was employed to expand this investigation. After interviewing those who met the inclusion criteria, they were asked to recommend another individual with similar conditions; thus, introducing the remainder of the participants. On the other hand, those who declined or refused to be audio-taped during their review were disqualified from taking part in this study.

As the participants were living in different cities in China, the network visual interview through the most popular Chinese social media application ‘WeChat’ by smartphone was used [28]. During the visual interviewing process, the interviewer and interviewee stayed in an independent, quiet, and safe environment of their home and office respectively.

### Data collection and analysis

To ensure reliability and validity throughout the research process, several steps were taken. Firstly, an initial interview outline was developed based on previous literature and existing theories related to the topic under investigation. In a pilot interview, all research contributors reviewed and updated the interview questions in another article, eventually leading to the formulation of an Interview Questions Guide (Table 1).

Then in April 2022, interviews were conducted with the eligible respondents respectively. Influenced by the isolation policy and different locations around China, interviewees all had one-on-one video interviews with researchers at their own homes (with no other family members present), and the duration of each respondent’s visit was adjusted according to his/her willingness to answer questions and provide valid information, around 60–90 min. At the beginning of each interview, some basic information about the respondent such as age, educational background, and years in the nursing profession was asked first; then core questions such as: What was your experience and feeling when facing the death of COVID-19 patients during your participation in medical support for Wuhan? How did you manage feelings and emotional changes related to patient death within a certain period? For any novel insights derived from each interview, the researchers employed a text annotation technique to standardize the unprocessed texts and explicitly categorize them based on their similarities and discrepancies. Subsequently, Graneheim and Lundman methods were utilized by the investigators for analysis and coding [25]. Though fourteen nurses were recruited, when the interviews reached fourteen people, theoretical saturation was observed and codes obtained from the interviews were repetitive, so we could not gain any new information. After ensuring that continuing the interviews would not form new codes, researchers terminated the interview process [29].

In addition, during the interview process, great attention was paid to the participant’s facial expressions, tones, and body language. After the interview, all of the content from it was read through thoroughly to understand its meaning; different independent concepts were compared multiple times with specific marks used to indicate their differences. The data was then coded according to similarities and differences into subcategories; ultimately themes were extracted by analyzing and interpreting this classified and summarized data.

To ensure the reliability and quality of the research results, this study followed Guba and Lincoln’s coding rules in sequence [30]. Careful examination was conducted to define the association between different topics or contents to timely discover hidden information and assign information appearing in texts to specific topics or contents. To ensure impartiality and prevent any personal bias from influencing the results of the coding and analysis, the authors sent their findings to some participants for review and held virtual meetings every 15 days to discuss their observations. This was used to verify whether the data obtained could accurately reflect their opinions and ideas. Two members of the research team (including the first author and corresponding author) jointly carried out data analysis. Comprehensive checks were made on all entered data for detecting any errors or residual problems. Taking into account the transferability of data, direct quotations from a large number of participants were adopted in this study for verification purposes apart from using their comments for naming subcategories; also direct use was made of participant’s quotations for a comprehensive description of that topic to make sure that no important details have been omitted during the analysis process.

**Table 1:** Interview Question Guide

NO.	Questions
1	What is your experience and feeling about the death of the COVID-19 patient?
2	How do you deal with the feelings and emotional changes related to the patient’s death?
3	Have your regular work and daily life been affected after the contact of the COVID-19 patient’s death?
4	What help and support do you most want to get when facing the patient’s death?
5	Concerning the nursing care of the dying COVID-19 patient, what are the parts you did well while others may have inadequate?

## Results

### Sample characteristics

This study described the experience of fourteen nurse participants with an age range of 23–41 years and 3–22 years of work experience, nine were married and six were mothers. The participants were from five provinces and cities in China: Hubei(Wuhan), Shanghai, Qinghai, Gansu, and Xinjiang. All directly cared for the COVID-19 patients and experienced the patient’s death at least once. Most had worked primarily as staff nurses on medical or surgical units; three were experienced, head nurses( Table 2).

After analyzing the data, four main categories were identified that are presented in Tables 1 and 3. psychological shocks related to COVID-19 patient’s death; 2. personal psychological adjustment and demands; 3. insights on life and values; and 4. need for relevant knowledge and skills. Examples with the original words of the participants follow.

### Theme 1: psychological shocks related to COVID-19 patient’s death

#### Sense of loss and helplessness

All fourteen nurses showed a strong sense of loss and helplessness for the patient’s death. COVID-19 is featured for its high contagion, rapid changes in the disease course, and no specific cure treatments. The two sample interview comments depict the great disappointment the nurses expressed about the sudden death of the patient.

‘The patient is so young, just as my age of 24. I tried my best during the rescue process and felt so sad about the failure of the rescue. I’m so sorry that I had done too little for her. It’s just about ten minutes, and young life is gone. I felt deeply powerless as a nurse, our ability is very limited (sigh).’(Participant 2).

‘It only took about half an hour for the old grandma to go from waking to coma. The disease of new coronary pneumonia is really dangerous and changes fast. I don’t want to recall these sad things anymore, it’s uncomfortable, very regrettable (low tone).’(Participant 12).

#### Sense of tension and fear

Six nurses mentioned the strong feeling of tension and fear caused by the quick death of the COVID-19 patient. They described the mood of nervous, fear, and even collapse caused by the rapid deterioration of the patient’s health and the sudden death, as well as their high exposure during the rescue process:

‘The novel coronavirus pneumonia is progressing very quickly. and I feel that the patient looked okay when he came, but the blood oxygen saturation got worse only after 1 night. Out of professional instinct, I just wanted to do my best during the rescue process. Afterward, I felt very scared and worried that I might get infected as I was highly exposed.’(Participant 4).

‘I feel that I am about to collapse. Even though I have the psychological preparation for a serious patient’s death, in those few days, one after another patient died, which made me cry. I felt scared of the disease. I’m afraid that I will encounter life-and-death problems again. I am afraid of going to work. I have never been so afraid of going to work.’(Participant 6).

#### Sense of sensitivity and anxiety

Half of the interviewees reported a strong sense of sensitivity and anxiety after experiencing the death of the novel coronavirus pneumonia patient:

‘I will still think of the patients’ faces and rescue scenes when I return to the hotel room off shift. It is hard to fall asleep. Even though I fell asleep, it’s very shallow.’(Participant 5).

‘Wearing an airtight protective suit, the work of caring for the patients is overwhelming. I felt my physiology reached its limit. Experiencing patients’ deaths make my emotion more sensitive. I feel anxious when I think that I may face dying patients tomorrow again.’ (Participant 11).

### Theme 2: personal psychological adjustment and demands

#### Personal psychological adjustment

Nurses mentioned that even though the deaths of COVID-19 patients seriously affected their work and life, their feelings were somewhat alleviated after personal adjustments like crying, self-reflection, talking with family members and friends to achieve social support from them, etc.:

‘I used to regard the cry as a fragile manner, but now I feel that crying is a reasonable expression of emotions. That’s a way to channel my emotions at the right time, so I could do a better job with my ongoing nursing care.’(Participant 1).

I am keeping in touch with my family members every day. Encouragement from families, friends, and colleagues made me feel refreshed. There are also many positive reports from the media which encouraged me and proud of myself as a Counter-marching nurse.

‘I reflect that I need to improve my professional and rescue knowledge and skills instead of being immersed in sadness. I adjusted my mood as more patients need me to take care of [them].’(Participant 9).

#### Psychological support demands

Some interviewees mentioned their hope that the hospital and public health institutes could provide psychological support like death education and online and on-site independent psychological consultations.

‘It would be nice to have a WeChat platform for personal-psychological counseling. We also need more knowledge and help on death education.’(Participant 8).

### Theme 3: insights on life and values

#### Cherish life and live in the moment

After experiencing the death of the COVID-19 patient, all interviewees said that they recognized that life was more precious and expressed life insight as a need to live in the moment.

After finishing the terminal treatment for the patient, I feel that life is very fragile and precious”. Participant 7: “This year’s novel coronavirus pneumonia makes me feel that it is a blessing to be alive.“

‘I feel that life is very fragile and precious after finishing the terminal treatment for the patient. COVID-19 makes me feel that it is a blessing to be alive.’(Participant 11).

‘Life is impermanent and short’ (long sigh, pause). I will not stay up late to play on the phone, early to bed, and early to rise and exercise 2 to 3 times a week. And I will try to influence my family members and friends to build a good lifestyle. (Participant 14)

#### Social responsibility and professional mission

Respondents all expressed a sense of social responsibility and a professional mission to support the prevention and control of this COVID-19 pandemic.

‘Although I know this disease is highly contagious, I can’t think of so much, I only want to do my best to save the patient during the rescue process. That’s the mission of nurses.’(Participant 6).

‘Do a good job in every shift. Try my best to meet the needs of each patient. I am worthy of my professional mission as a nurse. We must continue to fight the novel coronavirus pneumonia to the end. Victory is not far away, all the medical staff did a great job, and I feel quite magnificent (raised voice, excitement)’.(Participant 12).

#### Strengthening of professional value

The participants mentioned that nurses’ professional value has been sublimated by their self-recognition of their COVID-19 patient care during this challenge, and the positive social recognition of nurses also greatly strengthened their nursing values.

‘the novel coronavirus pneumonia is a huge disaster for all people. As nurses, we did a hard job, but I felt proud of my job. Our work let people all around see the value of nursing again ’(Participant 11).

‘All resources in the media praised the work of nurses. I saw a video of a cured patient on Douyin (Ticktock Chinese version). He said: those who said that the stars are the brightest cause they have never seen the nurse’s eyes. It’s so touching and made me cry. The spreading of this positive energy has strengthened my professional value in being a nurse. (Participant 14)

### Theme 4: needs for relevant knowledge and skills

#### Knowledge and skills of COVID-19 nursing

COVID-19 is a new contagious disease that broke out in humans as a global threat. Though the nurses received quick pre-work training, most participants still mentioned the lack of relevant knowledge and skills for frequently occurring new challenges at work.

‘There was only a hurried training of 2 days before entering the ward. When a patient asked about the efficacy and adverse reactions of the drug, I could not give an accurate reply in time, which negatively influenced my nursing work.’(Participant 3).

‘I am not very familiar with isolation protection skills and intensive care skills, especially the use of ECMO (extracorporeal membrane oxygenation) artificial lungs, and CRRT (continuous renal replacement therapy). (Participant 8)

#### Body care for infectious disease patients

Only four interviewees had experience in body care for infectious disease patients. Most nurses indicated that they did not have enough skills to after-care for infectious disease cadavers:

‘I work in a general ward before, and I had never taken care of infectious disease corpses. Though the procedures were included in the quick pre-training and materials shared in our We chat group, I still feel scared in the actual operation.’ (Participant 10).

#### Humanistic care and emotional support

The nurses also emphasized the need to provide mental and psychological support for dying patients and their families. Specific psychological care knowledge and skills of nurses need to be strengthened.

‘The patients have serious worries about death as they knew it’s a dangerous disease. But I can’t guarantee any treatment effect. I feel confused about how to provide the right psychological care.’ (Participant 1).

‘I am also very sad about the patient’s death, and I don’t know how to inform the families and support them (pause with a helpless look). I feel so pale and weak for any comfort.’ (Participant 13).

**Table 2:** Demographic characteristics of the nurses who took part in the study

Variable	Category	Frequency
Age	20–40	12
	> 40	2
Gender	Male	10
	Female	4
Education	Junior college	5
	Bachelor’s degree	8
	Master’s degree	1
Marriage status	Married	9
	Single	5
Working experience (years)	< 10	9
	10–20	3
	> 20	2
Work capacity/level	Senior level	1*
	Associate senior level	2*
	Intermediate level	2
	Junior level	9

*: Working experience as head nurse

**Table 3:** Categories, subcategories, and codes

Categories	Subcategories	Codes
Psychological shocks related to COVID-19 patient’s death	Sense of loss and helplessness	Acknowledging the powerlessness in not being able to save a patient’s life, as well as the sense of helplessness, sorrow, and deep disappointment often experienced.
	Sense of tension and fear	The feeling of being overwhelmed. The dread of going to work, having to confront the novel coronavirus, witnessing the passing away of critically ill patients, shedding tears for those who have passed on, often feeling tense and emotionally exhausted.
	Sense of sensitivity and anxiety	Experiencing the aftermath of death leads to difficulty falling asleep, sleep deprivation, emotional sensitivity, and anxiety due to work stress.
Personal psychological adjustment and demands	Personal psychological adjustment	Relieve negative emotions by crying, introspection, and discussing with family and friends.
	Psychological support demands	Death education and online and on-site independent psychological counseling are needed from hospitals or public health institutions.
Insights on life and values	Cherish life and live in the moment	Recognizing the transience of life, treasuring every moment, and feeling grateful for what we have; pursuing a healthier lifestyle by exercising regularly, getting plenty of rest, and eating nutritiously.
	Social responsibility and professional mission	Acknowledging the life-saving duty of nurses, their professional mission, and social responsibility.
	Strengthening of professional value	Recognition from society and self-identification elevate nurses’ professional values, significantly enhancing the nursing worth they bring.
Needs for relevant knowledge and skills	Knowledge and skills of COVID-19 nursing	The training is brief and unfamiliar, with a focus on intensive care work, potential side effects of certain medications, and the utilization of specialized equipment.
	Body care for infectious disease patients	Lack of experience in handling dead bodies of infected patients, resulting in fear and lack of nursing skills in the actual operation.
	Humanistic care and emotional support	The necessary support to alleviate the psychological distress of dying patients is often lacking, leaving families devastated by the loss of their beloved.

## Discussion

The study showed that the death of COVID-19 patients brought great psychological shock to the nursing staff, who needed psychological adjustment and adaptation. Personal regulation, family and social support, social responsibility, and professional values could enhance their transformation from negative emotions. The demand for relevant knowledge and skills such as dealing with the deaths of emerging infected persons was a new challenge for them.

This study shows that one of the most significant challenges nurses face is the psychological impact of patient death. Previous studies have demonstrated that when nurses are in close contact with patients suffering from emerging infectious disease, they can suffer from pain, loneliness, anxiety, fear, fatigue, sleep disorders, and other physical and mental health issues [31–33]. The results of this study revealed that most nurses still experienced considerable tension, fear, and anxiety after their patients passed away, which is consistent with the previous study. A cross-sectional report in 2020 on China found that nearly 70% and 50% of nurses experienced anxiety and despair, respectively, while caring for COVID-19 patients [34]. Kim et al. reported in 2018 that 22.2% of Korean nurses had posttraumatic stress disorder due to their care for MERS-CoV patients during the outbreak period. It is worth noting that although dealing with infectious diseases can bring additional pressure, new emerging infections often lack clear therapeutic drugs, so nurses not only have to pay attention to the needs and physical changes of the patient at all times but also bear a great sense of helplessness and loss when severe patients die without effective drugs. This feeling of helplessness may be different from the experience of death among ordinary patients [5, 35], accompanied by this powerlessness is worry about whether the nurses themselves will soon be infected and fear of death. Therefore, the responsibility of nurses as healthcare providers, in contrast to the need for safety as an individual, has increased their stress. In this study, a nurse (participant 2) mentioned that “our capacity is very limited and I am more worried about myself getting infected during caring for the died patient”.

Research has found that, in contrast to the past, during the pandemic prevention and control period, front-line nurses have focused on individual self-psychological adaptation and self-regulation for their entire psychological adjustment process. Although they had received unified psychological training and guidance at the beginning stage, it was short-lived and lacked relevant interventions from professionals. At the beginning of 2020, large numbers of front-line nurses were called up by governments to support Wuhan’s medical system, which was close to collapse due to massive population infection [36, 37]. As a result, they left their homes and families and were arranged for centralized accommodation with isolation management policies [28]. These factors all caused front-line nurses rarely have chances or time to seek face-to-face psychological support from family members, friends, or professional personnel. They often resorted firstly to physical catharsis such as crying and deep breathing; then looked for external spiritual supports such as watching positive media reports, video calls with family members, reading professional books, etc., but what they desired most was an authoritative platform with experts providing them full course one-on-one psychological consultation and help. Therefore, providing sufficient psychological support to nurses who have experienced patient death during the outbreak of an epidemic or pandemic is crucial for reducing their psychological trauma. It needs to be emphasized that such psychological damage may not be short-term and can likely affect the physical and mental health of nurses for a long period of time. A study on the long-term effects of SARS patients’ care reported that healthcare providers felt high levels of Post-traumatic stress disorder, even 13–26 months later [38]. Therefore, spiritual aid should focus on all stages of nurses’ mental condition development; Eftekhar et al.‘s recent study report on medical personnel working experience during the COVID-19 pandemic also showed that individual-centered interventions and effective maintenance should be implemented at each stage [39], which is consistent with the results of this study. On the other hand, managers should encourage nurses to vent their negative emotions when patients die, while following up a complete system of spiritual aid to meet the demands for psychological support from isolated nurses.

This study’s results also showed that nurses experienced growth in self-reflection, emotional stability, sense of social responsibility, and professional honor after caring for COVID-19 dying patients’ death. Both positive and negative emotions were present, which is consistent with the findings from multiple studies conducted in recent years [6, 39, 40]. It is widely accepted that psychological adaptation and social support play a mediating role in the whole process of systemic recovery under disease outbreak pressure [41]. In this study, nurses adopted self-reflection as well as avoidance of stress caused by death while strengthening their sense of social responsibility, enhancing nursing profession’s value, and maintaining hope to defend against and regulate psychological pressure. The results indicated that these coping measures could alleviate pressure and promote mental health for nurses during an epidemic or pandemic disaster. This was also supported by SARS ward nurses using various methods to cope with stress [42, 43]. Additionally, other results showed altruism, collectivism as well as solidarity spirit could better regulate individuals’ stress levels [44], which was also confirmed by this research result. Generally speaking, the nurse can adjust their cognitive rationality to adapt to the epidemic situation which may be related to healthcare professionals having medical knowledge as well as a more rational and positive attitude [6]. There are some ways that could be adopted to effectively manage the psychological pressures generated during an epidemic outbreak period for nurses; for example, nurses could continuously adjust their cognitive evaluation to promote balanced self-psychological development; seek collective support; keep an optimistic mentality to adapt to internal or external environment changes thus reducing physical or mental harm caused by pressures.

The Counter-marching Nurses were called up from all over the country and from various nursing specialties, with many of them not being specialist nurses but coming from general wards. They reported that they had not received sufficient knowledge preparation or skill training to deal with this emerging infectious disease of the respiratory system, especially how to care for the dying COVID-19 patients under the stressful isolated environment. It is also worth noting that previous evidence has highlighted a lack of nurse education on death [45–47]. In this study, most nurses need training on both humanistic care and emotional support for dead persons and their families as well as skills training on corpse preparation. Corpse preparation work is an extension of patient care for deceased patients which shows respect to patient dignity [48], so it is essential for managers to consider strengthening relevant training and health strategies on nursing death education. Additionally, team collaboration should be taken into account, such as arranging different levels, educational backgrounds and specialties within one nursing team. This would guarantee that nurses could better cope with sudden situations during an epidemic or pandemic period, increasing nurse’s adaptability and enhancing their professional skills through the collaborated team work; just like what one participant said: “It was fortunate that there were experienced nurses in our shift, they knew how to prepare corpses in the infectious disease ward or else I wouldn’t know what to do.”

## Limitations and Reflection

This study employed a qualitative research method to gain insight into the real experiences and emotions of Chinese Counter-marching nurses staff confronting the death of COVID-19 patients during the COVID-19 pandemic in Wuhan. It can provide valuable information for nursing managers and nurses themselves to understand the appropriate knowledge in order to formulate psychological intervention plans and training policies.

However, this study also has certain limitations. One of the major limiting factors is that participants did not have face-to-face interviews with researchers, and researchers could only observe patients’ body language through video to help understand participants’ semantics and true thoughts by magnifying facial expressions. Because most of the participants were interviewed at home, 2 nurses as mothers had to interrupt or pause in order to deal with children’s matters during the visit. Another drawback is that this study mainly applies to Chinese Counter-marching nurses in China cannot be applied to other countries with different cultures; it is suggested that similar studies be carried out in various nations and the outcomes be contrasted.

## Data Availability

The datasets generated and/or analyzed during the current study are not publicly available due to the restrictions of the funding support program but are available from the corresponding author upon reasonable request after the whole ongoing research is finished.

## Abbreviations

COVID-19: Corona Virus Disease of 2019
ECMO: extracorporeal membrane oxygenation.
MERS-CoV: Middle East Respiratory Syndrome Coronavirus
SARS: Severe Acute Respiratory Syndrome
