# Correction: Experience of Chinese counter-marching nurses with COVID-19 patients’ death in Wuhan: a qualitative study

**DOI:** 10.1186/s12912-024-02426-6

**Published:** 2024-10-11

**Authors:** Zhifang Guo, Kunli Wu, Huibin Shan, Younglee kim, Qilian He

**Affiliations:** 1 College of Nursing, Dali University, Dali, China; 2 Department of Infection Disease, Kunming Third People’s Hospital, Kunming, China; 3 People’s Hospital of Dali Bai Autonomous Prefecture, Dali, China; 4 Department of Nursing, College of Natural Science, California State University, San Bernardino, CA 92407 USA

**Correction: BMC Nursing (2023) 22:141**

10.1186/s12912-023-01270-4

The original article presents a minor typo in Table 2: Male should be 6, and Female should be 8.

